# The emerging role of irisin in experimentally induced arthritis: a recent update involving HMGB1/MCP1/Chitotriosidase I–mediated necroptosis

**DOI:** 10.1080/13510002.2022.2031516

**Published:** 2022-01-29

**Authors:** Rowida Raafat Ibrahim, Noha M. Shafik, Rasha Osama El-Esawy, Mervat H. El-Sakaa, Heba M. Arakeeb, Radwa Mahmoud El-Sharaby, Dina Adam Ali, Omnia Safwat El-deeb, Sara Ragab Abd El-Khalik

**Affiliations:** a Medical Biochemistry & Molecular Biology Department, Faculty of Medicine, Tanta University, Tanta, Egypt; b Pharmacology Department, Faculty of Medicine, Tanta University, Tanta, Egypt; c Physiology Department, Faculty of Medicine, Tanta University, Tanta, Egypt; d Anatomy & Embryology Department, Faculty of Medicine, Tanta University, Tanta, Egypt; e Clinical pathology department, Faculty of Medicine, Tanta University, Tanta, Egypt

**Keywords:** Arthritis, Irisin, high-mobility group box 1, necroptosis, Monocyte chemoattractant protein 1, RIPK1/3, ‌Chitotriosidase-1

## Abstract

**Objectives:**

Necroptosis is a tightly adjusted inflammatory necrotizing cell death signaling pathway that participates in pathogenesis of discrete diseases as rheumatoid arthritis (RA). Irisin is a myokine with immuno-modulatory effect. Evaluation of irisin efficiency as a novel therapeutic agent in experimentally induced RA via modulating immuno-inflammatory, necroptotic molecular and biochemical signaling pathways.

**Methods:**

RA was induced in 30 female Wister albino rats by a single subcutaneous injection of collagen-II with incomplete Freund’s adjuvant (CII-IFA) followed by booster immunization dose 10 days later. After 14 days of the injection, arthritis chronic phase was precipitated. 15 rats were treated by S.C irisin injection daily for 4 weeks. Joint tissue homogenate RIPK-3, MLKL, HMGB1, MCP1, IL-6, CHIT1, MDA, and PN levels were assessed calorimetrically. However, TNF-α mRNA expression level was evaluated by the qrt-PCR technique.

**Results:**

The results showed that irisin significantly decreases the level of all assessed biochemical parameters, except MDA, which was significantly increased in comparison with the correspondent values in the arthritic group with no treatment (ttt).

**Conclusions:**

Irisin exhibits therapeutic anti-inflammatory and antioxidant effects via modulating immuno-inflammatory, necroptotic molecular, and biochemical signaling pathways in experimentally induced RA in rats.

**Abbreviations:**

RA: rheumatoid arthritis; RIPK3: receptor-interacting protein kinase 1; MLKL: mixed lineage kinase domain-like protein; HMGB1: High-mobility group protein box 1; MCP1: Monocyte chemoattractant protein 1; IL-6: Interleukin 6; CHIT1: Chitotriosidase; MDA: Malondialdehyde; PN: Peroxynitrite; TNF-α: Tumor Necrosis Factor; qrt-PCR: quantitative real-time reverse transcription PCR; CII-IFA: collagen-II with incomplete Freund’s adjuvant; ttt: treatment

Note: *TNF-α* gene (NCBI GenBank Nucleotide accession # NM_012675.3); The housekeeping gene *GAPDH* (NCBI GenBank Nucleotide accession # NM_017008.4)

## 1. Introduction

Necroptosis is a tightly adjusted inflammatory necrotizing cell death signaling pathway [1]. It is distinguished by a specialized mechanistic route of activation, implicating a particular amyloid-like multiprotein complex known as necrosome [2]. This is achieved by receptor-interacting protein kinase 1(RIPK1) and 3 (RIPK3) invigoration either by auto- and/or trans-phosphorylation. RIPK3 attracts and phosphorylates mixed lineage kinase domain-like protein (MLKL) forcing it to be assembled in an oligomerized form that sparks plasma membrane rupture, causing a direct membrane bilayer disruption [3]. Those sequels coincide with a group of proinflammatory cytokines besides releasing cell damage–associated molecular patterns (DAMPs), advocating progressing inflammation with related tissue insults [4]. High-mobility group protein box 1 (HMGB1), one of the nonhistone chromatin-binding proteins, is discovered to exemplify an ideal DAMP molecule. Intracellular HMGB1 is expressed in all nucleated cells with the ultimate pivotal role in keeping physiological cellular homoeostasis, regulating gene expression, as well as guarding cells from hazardous and damaging oxidative stress influences [5]. Oppositely, the release of HMGB1 to extracellular space can flare immuno-inflammatory response through coupling with a group of receptors such advanced glycation end-products (RAGEs), nucleotide-binding oligomerization domain-like (NLRs) along with toll-like receptors (TLRs) [6]. However, necroptosis is evidently proved to be triggered by different stimuli in multiple pathological status, and tumor necrosis factor α (TNF-α) is supposed to be the best known necroptotic signaling pathway inducer via coupling to tumor necrosis factor receptor 1 (TNFR1) that is considered to be one of the death receptors [7]. Lately, necroptosis has been shown to play a role in the pathogenesis of a variety of disorders, including those with inflammatory features, neurodegenerative diseases, ischemic brain injuries, and liver and kidney injuries [8].

Rheumatoid arthritis (RA) is one of the above-mentioned diseases for which necroptotic cell death is implicated in their pathogenesis [9]. It is a long-lasting devastating autoimmune inflammatory disease of 1–2% of inhabitance. It affects multiple joints with concomitant systemic inflammation that is clinically manifested [10]. Rheumatoid arthritis–affected joints are infiltrated by leukocytes and inflammatory cells. The joints ache, become swollen, and then deteriorate into disfigurements with bone erosions, hence interfering with the human capability of movement [11]. Chemokines are a group of chemotactic inflammatory mediators released by infiltrated leucocytes in the synovial tissues and have more than fifty types [12]. Monocyte chemoattractant protein-I (MCP1) is one of the recently discovered chemokines that has selectivity for mononuclear phagocytosis and is expressed by various cell types such as leukocytes and fibroblasts [13]. Chitotriosidase (CHIT1, chitinase-1, EC: 3.2.1.14) is a chitinase enzyme member of 18 glycosyl hydrolases which is capable of digesting chitin substrates [14]. Activated macrophages mostly synthesize it to be involved in innate immunity. It is one of the main biochemical markers for not only inherited lysosomal storage disorders, but also many infectious diseases and chronic inflammatory conditions such as RA [15].

To date, no definite clinical therapy has been discovered for RA. Instead, all medications are directed to delay the progressive course and complication of the disease by many anti-inflammatory therapeutics that have multiple side effects [16]. So, there is a real need for a drug that targets RA underlying causative molecular and biochemical signaling pathways. ***Irisin*** is a small molecular mass skeletal muscle secreted myokine. It is a hydrolyzed by-product of membrane-bound fibronectin type III domain-containing protein 5 (FNDC5) extracellular domains [17]. It regulates energy balance, increases adipocyte Uncoupling Protein-1 (UCP1) expression, improves insulin sensitivity, and contains the spread of variable metabolic diseases, including obesity, type 2 diabetes mellitus (T2DM), and hypertension [18]. Irisin and other myokines have immunomodulatory actions, balancing pro- and anti-inflammatory states by influencing macrophage inflammatory response and increasing phagocytosis [19].

***The aim of this study*** was to examine the efficiency of irisin as a novel therapeutic agent in experimentally induced RA in rats via modulating immuno-inflammatory and necroptotic molecular and biochemical signaling pathways.

## 2. Materials and methods

### 2.1. Animals

Sixty experimental female Wister albino rats with an average weight of 180–220 g were obtained from the Tanta University animal house of faculty of medicine. They were subdivided randomly to be housed in clean cages, 5 rats/cage. They were left to be acclimatized for one week at an average temperature of 25 °C, with 12 h/12 h light dark cycle as well as free water and food access on demand. Experimental protocol was accredited from the Tanta University Faculty of Medicine Ethical Committee, according to the Care and Use of Laboratory Animals Guide of Institute of Laboratory Animal Resources, 1996.

### 2.2. Chemicals

All experimental study solvents and chemicals were of high analytical degree and were obtained from Sigma-Aldrich, St. Louis, MO, USA, unless other chemical sources were documented. Arthritis was induced by Collagen type II (CAS Number 9007-34-5) and incomplete Freund’s adjuvant that was constituted as a mixture of paraffin oil and mannide mono-oleate in a percentage of 85:15 (Catalog Number # F5506). Experimentally induced arthritic rat was treated by irisin (Catalog Number # SRP8039)

### 2.3. Experimental protocol

The rats were randomly categorized into four equal groups of fifteen rats per each group. The first group was called the **control group** that received no medications with free water and food access ad libitum. **The second group (referred to as the irisin-treated group)** received 150 µL of irisin solution (100 ng/mL distilled water) injected subcutaneously into the tail daily for 4 weeks [20]. In **the third group (referred to as the arthritic group)**, experimentally induced arthritis was elicited according to Trentham et al. [21] methodology that dissolves bovine collagen-II (CII) in 0.05 M acetic acid to be finally emulsified with incomplete Freund’s adjuvant to form 0.2 mL of total CII-IFA emulsification solution. The previously prepared CII-IFA emulsification solution was injected intradermally 2 cm away from the root at each rat’s tail. A booster immunization dose of 0.1 mL CII-IFA emulsified solution was subcutaneously injected into the tail’s opposite side 10 days after the first immunization. Experimentally induced chronic phase arthritis was obtained 14 days after the injection. **The final group (referred to as the irisin-treated arthritic group)** after induction of a chronic phase of arthritis, received 150 µL of prepared irisin solution (100 ng/mL distilled water) were subcutaneously injected in rat tail daily for 4 weeks [20]. A careful regular daily monitoring of periarticular tissues was accomplished to detect any affected joint swelling and erythema. The scores for each paw were then added to get the total arthritis score (maximum possible score 16 per animal, 4 for each paw according to the redness, swelling) and designated as the arthritic index on days 7, 14, and 21 according to the arthritis scoring system [22].

### 2.4. Tissue sampling

At the end of the experiment, all rats were sacrificed by decapitation after anesthesia. After decapitation, the backward knee joints were dissected carefully, washed three times in ice cold saline, and weighed and divided into three sections. The first sample was kept in 10% v/v buffered paraformaldehyde solution for further histopathological examination. The second sample was used to prepare tissue homogenates, and finally, the third sample of excised knee joint was kept at −80°C for gene expression evaluation.

### 2.5. Knee joint tissue homogenization

Isolated tissue from knee joints (200 mg) were placed onto dry ice and ground to a powder using a mortar and pestle. Next, the material was warmed to room temperature and a suspension was prepared by adding PBS (1 mL, 50 mM, pH 7.4). The suspension was diluted to a final volume of 25 mL with PBS containing one tablet of complete TM Protease Inhibitor Cocktail [23] and homogenized for 20 min with a Potter-Elvehjem homogenizer (Sigma-Aldrich, USA). The joint tissue homogenate was centrifuged (12,000 rpm) for 10 min at 4°C. The supernatant was collected in centrifugation tubes to be centrifuged again (3000 rpm) for 5 min. The final resultant supernatant was stored in sterilized eppendorf at −80°C for biochemical marker assessment. Joint tissue protein concentrations were quantitated by the Lowery method [24].

### 2.6. Biochemical analysis

#### 2.6.1. RIPK3 and MLKL

RIPK3 and MLKL levels were determined in joint tissue homogenates by using the sandwich enzyme–linked immunosorbent assay (ELISA) kit (Sun-red Biological Technology Co, Shanghai, China, Cat# 201-11-1781) and Life Span BioSciences, Inc. Cat# LS-F55370, respectively, according to the manufacturer’s instructions. Optical densities of the samples were then measured at 450 nm on Stat Fax 2000 ELISA reader. The standard curves of known RIPK3 and MLKL concentrations were established and employed to determine RIPK3 and MLKL concentrations, respectively, within the samples. The results were expressed as ng/mg protein.

#### 2.6.2. HMGB1, MCP1, and IL-6 levels

In joint tissue, homogenates were quantitatively assayed following the manufacturer’s instructions of the enzyme-linked immunosorbent assay (ELISA) kits (My BioSource, Inc. Southern California, San Diego (USA) Catalog No: MBS703437, MBS2506535 and MBS355410, respectively). The color intensities were read at 450 nm on Stat Fax 2000 ELISA reader. The **HMGB1** and **IL-6** values were expressed as pg/mg protein. However, the levels of **MCP1** were expressed in unit ng/mg protein.

#### 2.6.3. Quantitative measurement of tumor necrosis factor α (TNF-α) gene expression by quantitative real-time reverse transcription PCR (qrt-PCR)

- *RNA extraction:* According to the context of the protocol from the manufacturer and as described before [25], total RNA was extracted from Rat backward dissected knee joint cells using the Gene JET RNA Purification Kit (Thermo Scientific, #K0731 USA). By using a NanoDrop spectrophotometer (NanoDrop Technologies, Inc. Wilmington, USA), the total RNA concentration and purity were measured at the OD260 and OD260/280 ratios, respectively, and RNA was then preserved at−80°C.
- *cDNA synthesis:* Revert Aid H Minus Reverse Transcriptase (Thermo Scientific, # EP0451, USA) was utilized to reverse-transcribe the total RNA samples (5 µg), producing cDNA that was stored at −20°C to be used for PCR.
- *Quantitative real-time PCR:* cDNA was used as a template for determining the relative expression of the **TNF-α** gene by using StepOnePlus real-time PCR system (Applied Biosystem, USA). By Primer 5.0 software, the above-mentioned gene primers were designed and their sequences were as follows: **TNF-α (NCBI GenBank Nucleotide accession # NM_012675.3)** F: 5′- AGAACTCCAGGCGGTGTCTCTG -3′, and R: 5′- GTGGCAAATCGGCTGACGGTGT-3′. The housekeeping gene **GAPDH** with primer sequences **(NCBI GenBank Nucleotide accession # NM_017008.4)** F: 5′-ATGTTCCAGTATGACTCCACTCACG-3′ and R: 5′ GAAGACACCAGTAGACTCCACGACA-3′ was used as a reference for fold change in target gene expression calculation. A final volume of 25 µL PCR mix was prepared by adding 12.5 µL of 2X Maxima SYBR Green/ROX qPCR Master Mix (Thermo Scientific, # K0221, USA), 2 µL of cDNA template, 1 µL forward primer, 1 µL reverse primer, and 8.5 µL of nuclease-free water. The thermal cycling conditions were as follows: Initial denaturation at 95°C for 10 min was followed by 40 cycles with denaturation at 95°C for 15 s annealing at 60°C for 30 s, and extension at 72°C for 30 s. At the end of the last cycle, the temperature was increased from 60 to 95°C for melting curve analysis. The relative expression level of genes was normalized to GAPDH and analyzed using the threshold cycle (Ct) 2^−ΔΔCt^ method [26].

#### 2.6.4. Chitotriosidase (CHIT1) enzyme activity

Chitotriosidase (CHIT1) enzyme activity was assayed spectro-fluorometrically in joint tissue homogenates [27] depending on the synthetic substrate 4-methylumbelliferyl-β-D-NN, N'- triacetylchitotriose enzymatic hydrolysis by **CHIT1**, resulting in fluorescent molecule 4-methylumbelliferone (4MU) release. This can be measured fluorometrically at 365 nm excitation wavelength and 450 nm emission wavelength. The emitted fluorescence is proportional to the amount of the hydrolyzed molecules.

#### 2.6.5. Malondialdehyde (MDA)

MDA level in joint tissue homogenates was spectrophotometrically carried out according to the previously described method [28], where 250 µl of homogenate were shaken with 1.25 mL of trichloroacetic acid (TCA) and 0.5 mL of thiobarbituric acid (TBA) (0.33% w/v) was added to this fraction. All tubes were heated for one hour at 95°C in a water bath. After cooling, the TBA-MDA complexes were extracted with 2 mL of N-butanol. The resultant N-butanol layer was separated and the absorbance was determined at 532 nm. The light absorbance was read at 532 nm on a spectrophotometer and MDA levels were determined from a standard curve that was generated from 1,1,1,3 Tetramethoxypropane. The results are represented as nmoles/mg tissue.

#### 2.6.6. Peroxynitrite (PN) level

Peroxynitrite (PN) level in joint tissue homogenates was determined under guidance. 10 µL of homogenate was added to 5 mM phenol in 50 mM sodium phosphate buffer to a final volume of 2 mL and mixed well, and then incubated for 2 h at 137 °C. A total of 15 µL of 0.1 NaOH were added and mixed. The absorbance of the sample was recorded at 412 nm by using a spectrophotometer. Peroxynitrite was calculated using ϵ = 4400 M^−1^ cm^−1^ as a molar extinction coefficient [29].

### 2.7. Histopathological and immuno-histochemical examination

The backward knee joints specimens were kept in 10% v/v formalin solution for 24 h, washed by tap water for half an hour, and then the specimens were decalcified in the chelating agent disodium EDTA solution 4% w/v (Drury and Wallington, 1980) [30]. Decalcification lasted for about 4 weeks, during which the solution was renewed every 2 days until the tissues had softened. The decalcified knee joints were cleaved longitudinally in a sagittal plane along the central portion, and specimens were processed to form paraffin blocks. The fixed specimen was dehydrated after washing in ascending grades of ethyl alcohol for one hour for each as follows: 50%, 60%, 70%, 80%, 90%, and two times in 100% v/v (absolute alcohol). Then, the specimens were cleared with xylene and then for 15 min for each impregnated and embedded in paraffin at 60 °C. The blocks were cut into sections 5 microns thick by using a rotatory microtome (Leica SM 200R), were placed on numbered glass slides, and dried overnight in an oven at 37 °C. After this, the slides were immersed in two changes of xylene for 15 min for each and then were placed for 1 min in each of four changes of absolute alcohol. Consequently, the slides were placed in descending grades of alcohol from 90% and 70% to 50% v/v and then were immersed in distilled water. The sections were subjected to the following stains:

#### 2.7.1. Hematoxylin and eosin [H&E]

The sections were stained in hematoxylin for 15 min and washed in tap water for 10 min. Then they were stained in eosin for 1 min and then mounted and covered to protect them before examination [30].

#### 2.7.2. Immune staining using anticollagen type II antibody (2B1.5) mouse monoclonal antibody (Lab Vision Co)

This Immune staining was used [31] to assess the relative proportions of collagen II in the regenerated tissue. The specimens were diluted at (1:20 dilution) at 4 °C overnight. Localization of collagen fibers at the matrix of chondrocytes was visualized using a biotinylated secondary antibody and the conventional avidin–biotin-peroxidase method with diaminobenzidine (DAB) as the substrate. Then, the slides were counterstained with Mayer’s Hematoxylin before mounting [32].

#### 2.7.3. Toluidine blue for proteoglycans

The slides were immersed in 0.04% v/v Toluidine blue for 5–10 min, rinsed with tap water for 1 min, dried with warm air for 9 min, cleared with xylene for 5 min, and then mounted with resinous mounting media and covered [33].

### 2.8. Statistical analysis

Data analysis was achieved using GraphPad Instat software (Version 2.0 Philadelphia, 1993). All data were tested by the Kolmogorov–Smirnov Goodness of Fit Test (K-S test) as a test for normality and the data were normally distributed. The data were expressed as mean ± SD. Comparisons were done using one-way ANOVA followed by Tukey–Kramer as a post ANOVA test. The criterion for significance was chosen to be at *p* ≤ 0.05.

## 3. Results

### 3.1. Effect of irisin on arthritic index A.I (Figure 1)

**Figure 1.**
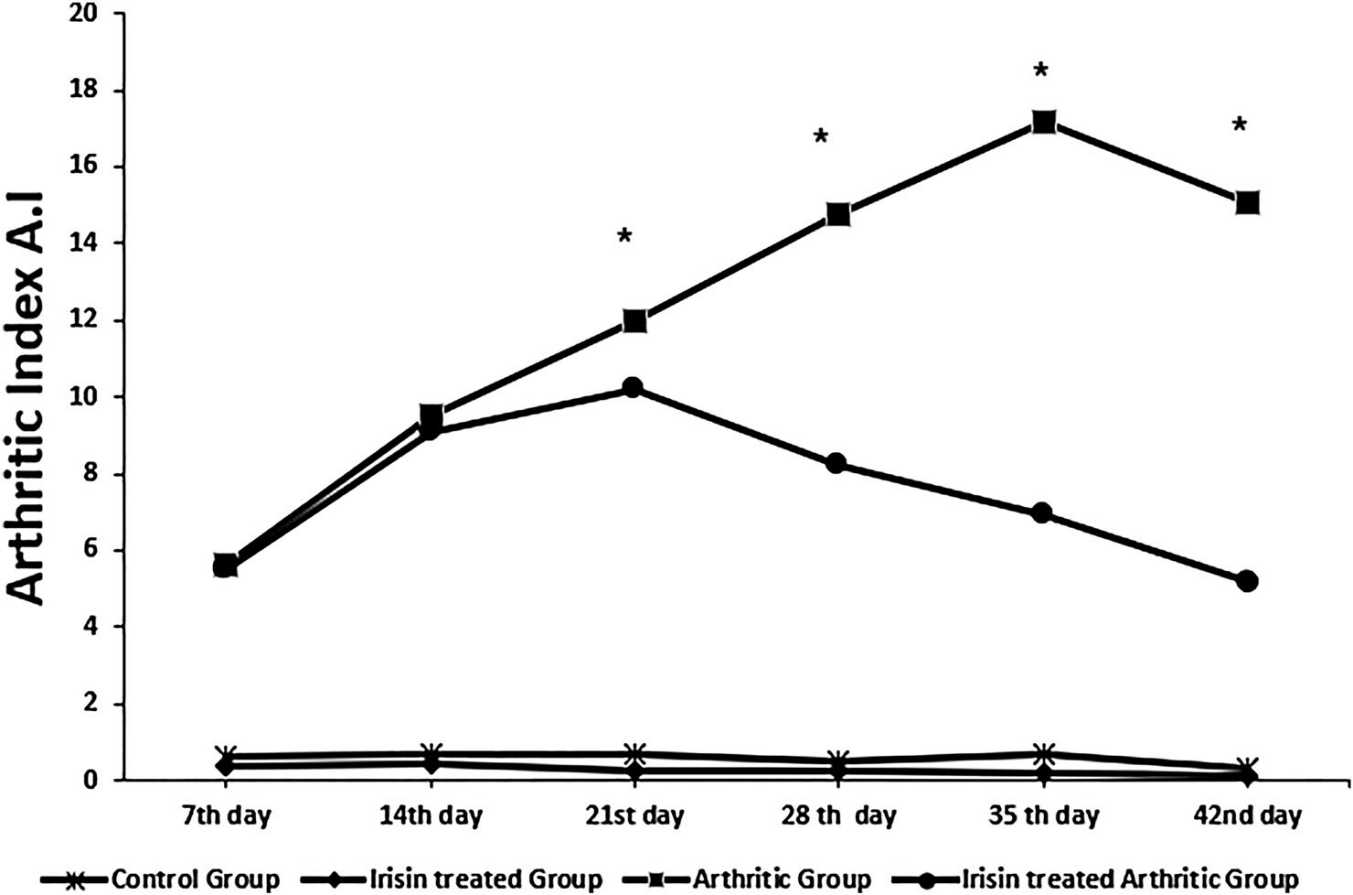
Arthritic indices A.I among the all the studied groups over a 6-week experimental duration. *significance (*p* ≤ 0.05). *Arthritis index is a score that is used to evaluate the degree of inflammation. Score each foot claw, 0–4 points in the record, and the cumulative score is the arthritis index of each mouse.* [55].

As illustrated in Figure 1, the A.I is significantly increased along the course of the study every week to reach its maximum level at the 35th day in the arthritic group. However, upon irisin ttt in the irisin-treated arthritic group, A.I values started to significantly decrease when it was statistically compared with the arthritic group from the 21st day to reach its lower level at the end of the experiment (42nd day), and this last A.I level was not significantly different from the 7th day A.I value of the same group.

### 3.2. Effect of irisin on necroptotic signaling biochemical pathway RIPK3 and MLKL (Tables 1 and 2)

Table 1 demonstrates that although there were no significant differences of both RIPK3 and MLKL values between both control and irisin ttt groups, both RIPK3 and MLKL values were significantly increased in the arthritic group. In contrast to the arthritic group, the levels of RIPK3 and MLKL decreased significantly in the irisin-treated arthritic group, with still a significant difference between RIPK3 and MLKL and both control and irisin ttt groups for RIPK3. In addition, both RIPK3 and MLKL values showed significant positive correlation with A.I, HMGB1, MCP1, IL-6, CHIT1, and oxidative stress biomarker (MDA and PN) levels in both arthritic group and irisin-treated arthritic group as clarified in Table 2.

**Table 1.** Shows a comparison of the arthritic index, joint tissue homogenate RIPK3, MLKL, HMGB-1, MCP-1, IL-6, CHIT1 activity, MDA, and PN levels among the studied groups using ANOVA test.

	Control group (*n* = 15)	Irisin-treated group (*n* = 15)	Arthritic group (*n* = 15)	Irisin-treated arthritic group (*n* = 15)	ANOVA	
					F	*P*
RIPK3 ng/mg protein	1.74 ± 0.37	1.56 ± 0.24	6.13 ± 1.34 ^a,b,d^	2.72 ± 0.39 ^a,b,c^	125.8	<0.001*
MLKL ng/mg protein	2.22 ± 0.45	1.62 ± 0.44	7.32 ± 1.43 ^a,b,d^	2.96 ± 0.19^b,c^	164	<0.001*
HMGB-1 pg/mg protein	7.74 ± 0.6	7.07 ± 0.76	19.7 ± 2.21 ^a,b,d^	8.83 ± 1.24 ^b,c^	287.8	<0.001*
MCP-1 ng/mg protein	2.05 ± 1	1.92 ± 0.65	14.3 ± 2.3 ^a,b,d^	3.15 ± 0.75 ^c^	296.4	<0.001*
IL-6 pg/mg protein	360 ± 9.5	336 ± 27.2	768 ± 39.4 ^a,b,d^	379 ± 13.3 ^b,c^	991	<0.001*
CHIT1 mmol/mg.ptn/h	28.3 ± 4.8	25.6 ± 3.8	74.7 ± 8.7 ^a,b,d^	34.1 ± 6.2 ^b,c^	209.7	<0.001*
MDA nmoles/mg tissue	9.7 ± 0.4	9.4 ± 0.4	67.1 ± 6 ^a,b,d^	12.3 ± 2.4 ^c^	1143	<0.001*
PN µmol/mg.ptn	15.7 ± 2.53	13.6 ± 2.18	32.8 ± 2.36 ^a,b,d^	21.5 ± 2.56^a,b,c^	189.9	<0.001*

Note: n: number of rats in each group; RIPK3: receptor-interacting protein 3; MLKL: mixed lineage kinase domain-like protein; HMGB-1: High-mobility group protein box 1; MCP-1: Monocyte chemoattractant protein-I; IL-6: interleukin 6; CHIT1: Chitotriosidase enzyme activity; MDA: malondialdehyde; PN: peroxynitrite; Data presented as mean ± SD, *significance (*p* ≤ 0.05), ^a^significant difference in comparison with the control group, ^b^significant difference in comparison with the irisin-treated group (II), ^c^significant difference in comparison with arthritic group III, and ^d^significant difference in comparison with irisin-treated arthritic group IV.

**Table 2.** Shows Pearson’s Correlations (r) between the studied parameters in the arthritic group (group III) and the irisin-treated arthritic group (group IV).

	Arthritic group (Group III)				Irisin-treated arthritic group (Group IV)			
	A.I	RIPK3	MLKL	HMGB-1	A.I	RIPK3	MLKL	HMGB-1
RIPK3	0.870*				0.766*			
MLKL	0.828*	0.927*			0.748*	0.917*		
HMGB-1	0.734*	0.945*	0.888*		0.663*	0.801*	0.747*	
MCP-1	0.753*	0.896*	0.780*	0.831*	0.801*	0.960*	0.866*	0.905*
IL-6	0.944*	0.941*	0.947*	0.865*	0.695*	0.947*	0.907*	0.684*
CHIT1	0.897*	0.964*	0.951*	0.928*	0.585*	0.807*	0.773*	0.714*
MDA	0.893*	0.953*	0.942*	0.925*	0.655*	0.935*	0.912*	0.717*
PN	0.886*	0.959*	0.951*	0.932*	0.662*	0.861*	0.839*	0.898*

Note: A.I: Arithritic index; RIPK3: receptor-interacting protein 3; MLKL: mixed lineage kinase domain-like protein; HMGB-1: High-mobility group protein box 1; MCP-1: Monocyte chemoattractant protein-I; IL-6: interleukin 6; CHIT1: Chitotriosidase enzyme activity; MDA: malondialdehyde; PN: peroxynitrite, *significance (*p* ≤ 0.05).

### 3.3. Effect of irisin on joint tissue homogenate HMGB1, MCP1, proinflammatory cytokines and CHIT1 (Tables 1 and 2)

Table 1 demonstrates that HMGB1, MCP1, IL-6, and CHIT1 values were slightly insignificantly decreased upon irisin treatment in the irisin ttt group when compared with that in the control group. But HMGB1, MCP1, IL-6, CHIT1 values were significantly increased in the arthritic group. Upon irisin ttt in the irisin-treated arthritic group, HMGB1, MCP1, IL-6, CHIT1 values were significantly decreased when compared with that in the arthritic group with still a significant difference between HMGB1, MCP1, IL-6, CHIT1, and irisin ttt group, except for MCP1. Also, the above-mentioned parameter values showed significant positive correlation with A.I. in both groups, with additional significant positive correlation between HMGB1 and MCP1, IL-6, CHIT1, MDA, and PN levels in both groups as presented in Table 2.

### 3.4. Effect of irisin on joint tissue homogenate relative TNF-α mRNA gene expression level: (Figure 2)

**Figure 2.**
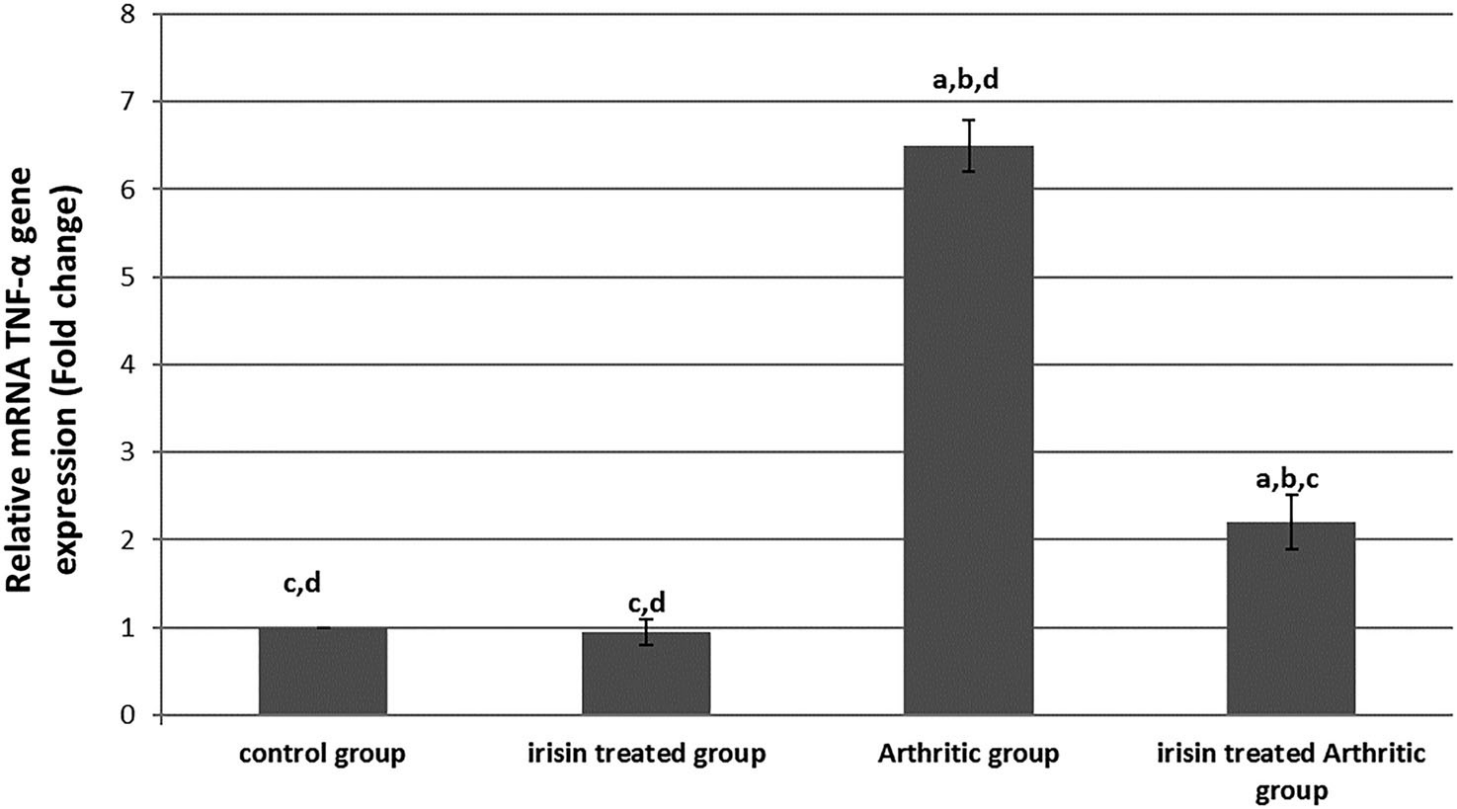
Quantitative real-time PCR analysis of the expression of TNF-α gene in different groups, values expressed by fold changes ± SD, ^a^significant difference in comparison with the control group, ^b^significant difference in comparison with the irisin-treated group (II), ^c^significant difference in comparison with the arthritic group, and ^d^significant difference in comparison with the irisin-treated arthritic group.

Figure 2 emphasizes that there was a significantly increased TNF-α mRNA gene expression in the arthritic group of up to about 6.5-fold changes to its level of expression in the control group. But irisin significantly decreased its level of expression in the irisin-treated arthritic group to only 2.2-fold changes to its level of expression in the control group, which was still significantly higher than in the control and irisin ttt groups.

### 3.5. Effect of irisin on oxidative stress status (Table 1)

Both MDA and PN levels were significantly markedly increased in the arthritic group when compared with that in the other groups. But MDA and PN values showed a significant decrement upon irisin ttt in the irisin-treated arthritic group and at the same time were still significantly higher than MDA and PN values in both control and irisin ttt groups, as shown in Table 1.

### 3.6. Histopathological examination (Figures 3–5)

**Figure 3.**
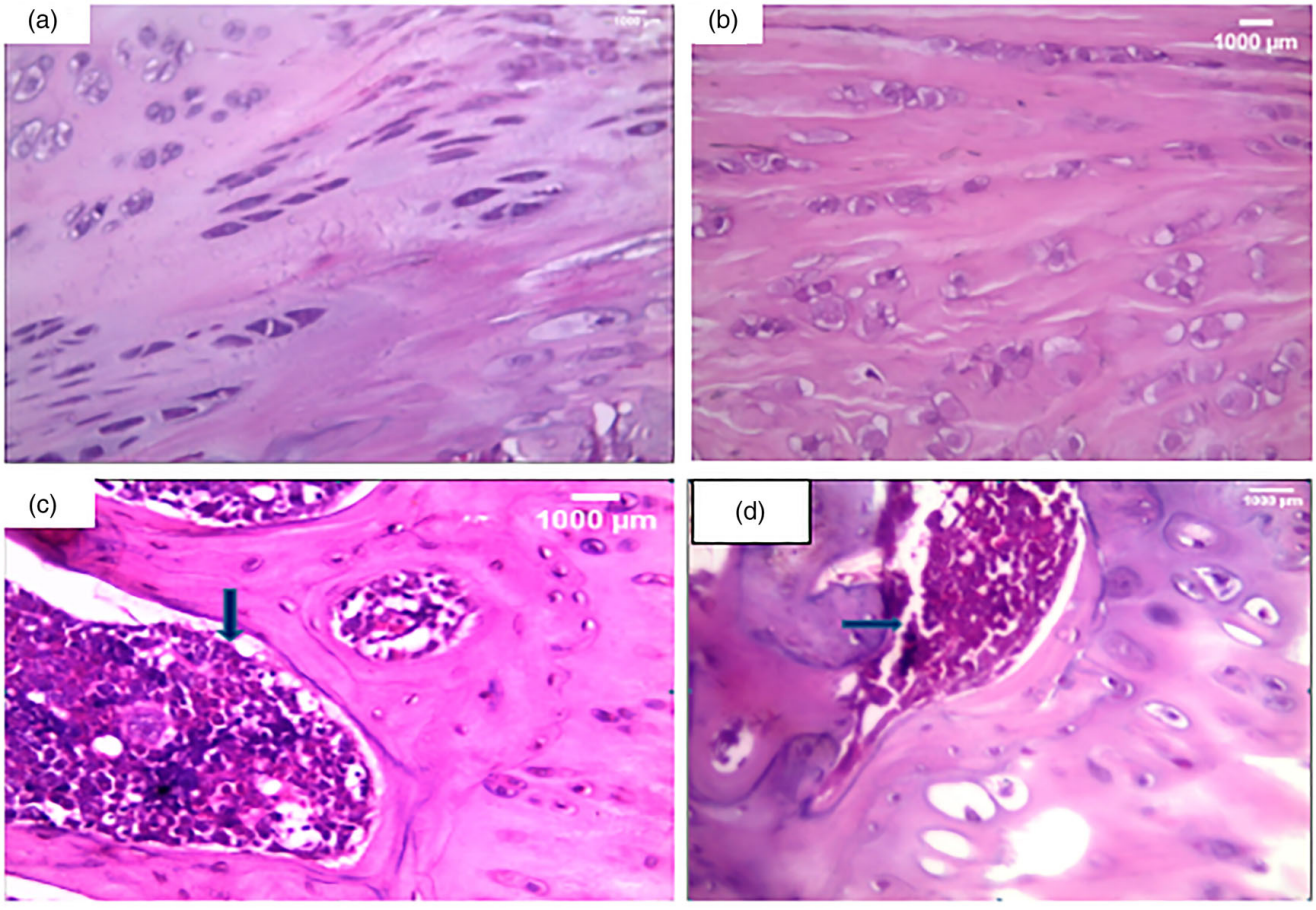
Histopathological examination of knee condyle articular cartilage sections: (a): A section in the articular cartilage of a knee condyle of the normal control group showing normal cartilage. (H&F X400), (b): A section in the articular cartilage of a knee condyle of the irisin-treated group showing normal cartilage with no inflammatory cellular infiltrate … (H&E X400), (c): A section in the arthritic group showing a dense lymphoplasmacytic infiltrate that extends to the subchondral bone illustrated by the blue arrow. (H&E X400), (d): A section of the irisin-treated arthritic group showing a moderate inflammatory cellular infiltrate, illustrated by the blue arrow. (H&E X400).

Histological examination (Figure 3) revealed a smooth regular articular surface with normal thickness of all zones with a near absence of inflammatory cellular infiltrate (Figure 3(A,B)) in both control and irisin ttt groups. However, there was a disturbed irregular surface, an irregular distribution of chondrocytes, and a dense lymphoplasmacytic inflammatory cellular infiltrate that extended to subchondral bone (Figure 3(C)) in the arthritic group. Finally, there was an incomplete restoration of the cartilage surface with a moderate inflammatory cellular infiltrate in the irisin-treated arthritic group in comparison with normal articular cartilage (Figure 3(D)).

**Figure 4.**
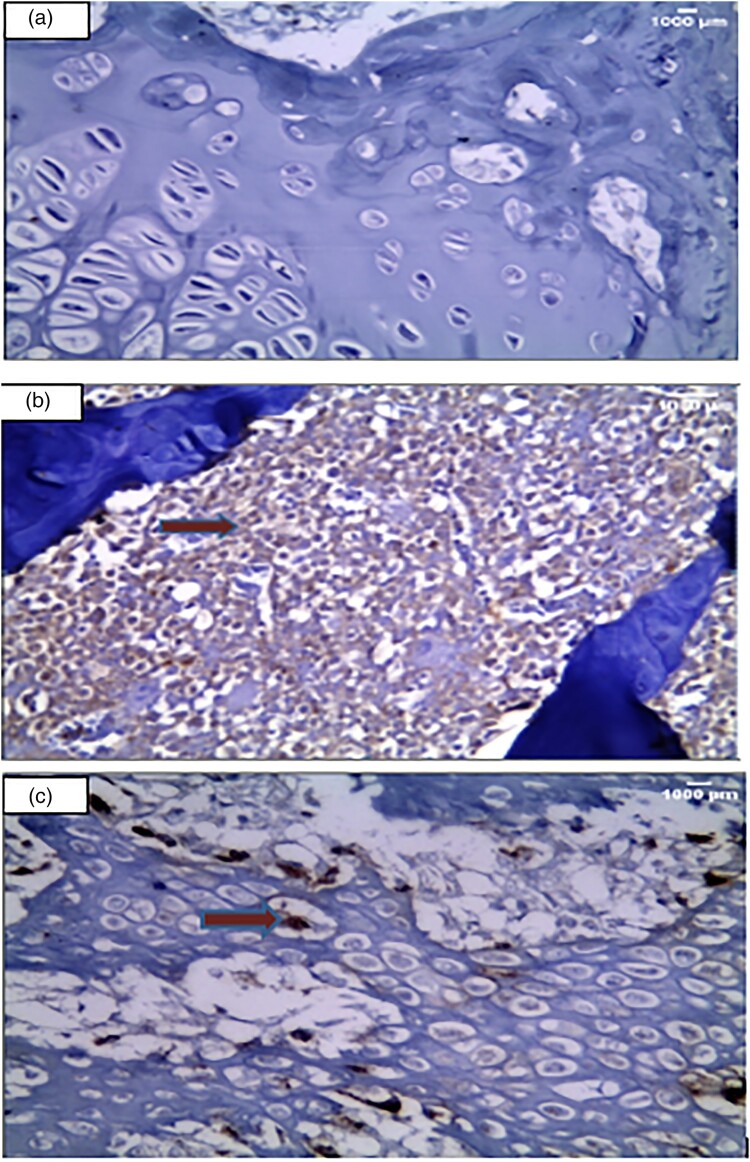
Immuno-histochemical examination (Anti- collagen II x 400). (A): A section in the articular cartilage of knee condyles of the control group showing a negative expression of anti-collagen II in the control group (X400) (B): The knee articular cartilage of the arthritic group showing a strong diffuse anticollagen II expression in inflammatory cells, illustrated by the red arrow in the arthritis group (x400) (C): Section in the irisin-treated arthritic group knee condyles showing a focal moderate expression of anti-collagen II, illustrated by the red arrow (X400).

Immuno-histochemical examination (Figure 4) showed negative expression for anti-collagen II stain in the matrix and in the small inflammatory cellular infiltrate in the control group (Figure 4(A)). Whereas there was strong diffuse expression in the inflammatory cellular infiltrate at the site of arthritis in the arthritic group (Figure 4(B)). As well, there was moderate and focal expression for the anti-collagen II stain in the articular cartilage showing a few brownish discolorations of the chondrocyte matrix at the site of arthritis after irisin treatment in the irisin-treated arthritic group (Figure 4(C))

**Figure 5.**
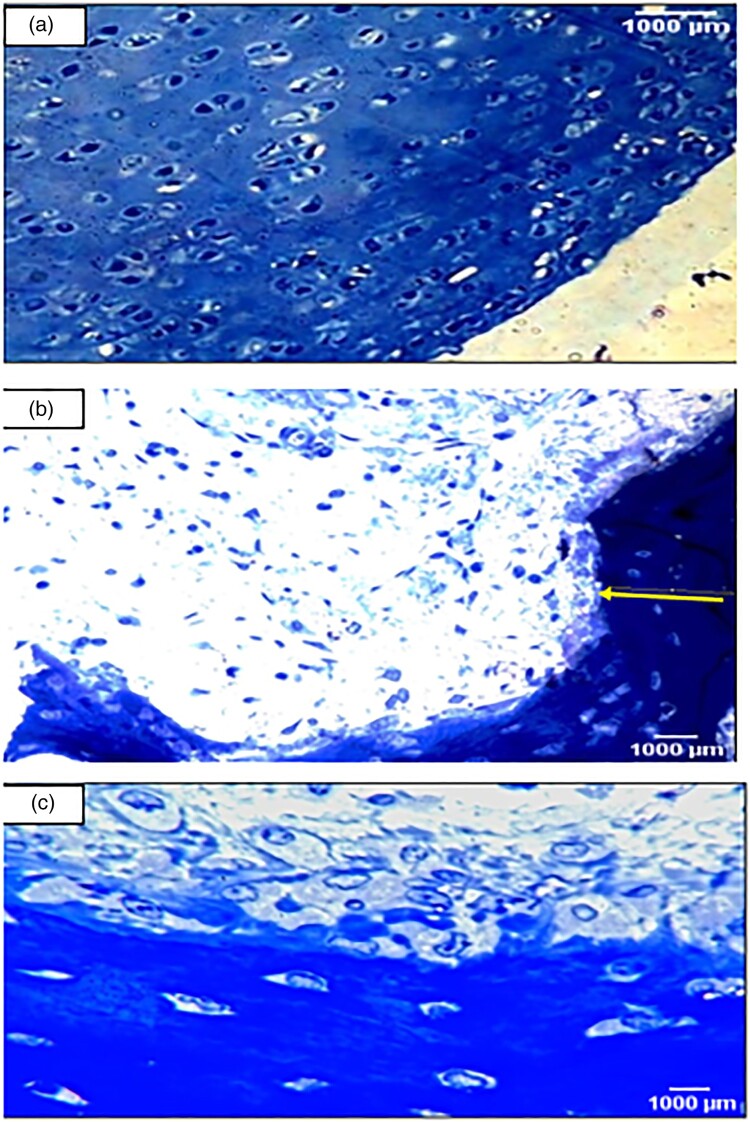
Toluidine blue staining examination (Toluidine blue x 400). Cartilage matrix and nuclei appear as deep violet. However, cytoplasm and other tissue elements are shown light blue. (a): A section in the articular cartilage of knee condyles of the control group showing normal thickness of the cartilage [toluidine blue X400] (b): The knee articular cartilage of the arthritic group showing a decrease in thickness of the cartilage [toluidine blue X400] (c): Section in the irisin-treated arthritic group showing knee condyles with a moderately increased thickness of the cartilage compared with that in the arthritic group [X400].

Toluidine blue staining of the control group showed normal staining of the cartilage matrix and cellular cytoplasm (Figure 5(A)). On the other hand, there was a full thickness cartilage defect with a total loss of toluidine blue staining in the arthritic group (Figure 5(B)). However, in the Irisin-treated arthritic group, there was a moderate increase of cartilage thickness compared to that in the arthritic group (Figure 5(C)).

## 4. Discussion

Rheumatoid arthritis (RA) is a progressive autoimmune polyarthritis disease characterized by severe bone loss and inflammatory cell infiltration in the affected joints [34]. Recent evidence clarified the role of necroptosis as a downstream target of massive inflammatory process taking place in RA and a critical regulator of inflammatory cell death [35]. Indeed, the expression of necroptosis mediators RIPK1, RIPK3, and MLKL was significantly increased in the synovium and joint tissue of mice and RA patients. Therefore, RIPK1 and MLKL have proved to be efficient therapeutic targets in various inflammatory diseases, including RA [36]. TNF-α, a pleiotropic cornerstone mediator of inflammation, tissue injury, and cell death in RA, is thought to be the primary inducer for necroptosis [37]. After the binding of TNF-α to its receptor, TNFR1 undergoes a conformational change that recruits multiple proteins to form complex I, consisting of TRADD, RIP1, and several other elements. Then, RIP1 binds to RIP3, and the latter, in turn, recruits and phosphorylates MLKL to form necrosome. The phosphorylated MLKL is then translocated from the cytosol to the plasma and intracellular membranes. The oligomerization of MLKL results in membrane pore formation, inducing membrane rupture and eventually necroptosis [38]. Additionally, the induction of ROS and calcium-induced lysosomal membrane permeabilization (LMP) has been suggested to critically participate in the execution of programmed necrosis to disrupt cell integrity [39]. The aforementioned findings were in agreement with our results as evidenced by the significant increase in the levels of joint tissue homogenate RIPK3, MLKL, in addition to increased gene expression of TNF-α in the CII-IFA-induced arthritic group as compared with that in the other groups.

Presently, a large body of evidence has placed HMGB1 in a central position in the pathogenesis of RA [40]. Recent studies have proved that necroptosis is accompanied by a release of nuclear HMGB1 into extracellular space and this secreted HMGB1 markedly and extensively activates necrotic cells during inflammatory responses, resulting in further amplification of the inflammatory state through augmenting the genetic expression of MCP1 as well as IL-6 [41]. Notably, aberrant extranuclear HMGB1 expression in RA occurs in the serum, synovial tissue, and synovial fluid of RA patients [42]. These previous outcomes are in complete agreement with our results herein as evidenced by the significant increase in HMGB1 in the CII-IFA-induced arthritic group as compared with that in the other groups. Chitotriosidase-1 (CHIT1) has been recently considered as a highly sensitive biomarker for RA disease activity, contributing to the pathogenesis of RA through enhancing inflammatory and tissue remodeling processes [43]. These data are in accordance with our results as shown by the marked elevation of joint tissue CHIT levels in the CII-IFA-induced arthritic group as compared with that in the other groups.

Irisin is a recently discovered myokine that is released by skeletal muscle and adipose tissue. Recent research studies have focused comprehensively on its pleiotropic impact. Irisin has been revealed to have considerable impacts on body metabolism and thermogenesis. Surprisingly, serum irisin levels were much lower in RA patients. This lower serum irisin level was associated with higher RA activity, extraarticular manifestations, and greater class of failure of functional joints [44,45]. Irisin mediates a cross talk between muscle and cartilage by targeting chondrocytes, promoting glycosaminoglycans content and increasing collagen Type II gene expressions through the inactivation of p38/ERK MAP kinase signaling cascades, resulting in effective chondrocyte recovery [46]. In the present study, the irisin-treated arthritic group exhibited a remarkable reduction in necroptotic signaling biochemical pathway markers as compared with the CII-IFA-induced arthritic group. This could be attributed to a marked abrogating effect of irisin on TNF-α mRNA expression along with the mitigating effect of MCP1 and HMGB1 on downstream targets. In accordance with our results, irisin’s remarkable anti-inflammatory effect was mentioned by Dong et al. [47], who noted a reduced gene expression of proinflammatory markers TNF-α, IL-1B, and IL-6 in the muscle of mice and adipose tissues through the promotion of an alternative polarization of macrophages from M1 to M2 types. Irisin was also reported to suppress proinflammatory activation of macrophages [48] as well as NF-kB activation in malignant breast epithelial cells [49]. Moreover, irisin significantly decreased the TLR4 and MyD88 protein levels, as well as the phosphorylation of NF-kB, consequently leading to a reduction in the release of crucial pro-inflammatory mediators, including TNF-α, MCP1, and HMGB1 [50]. Furthermore, irisin efficiently mediated the hepatoprotective effects of dexmedetomidine on inflammation, and neutrophil infiltration, by inhibiting NLRP3 inflammasome activation in an intestinal ischemia/reperfusion experimental model [51].

Olson et al. revealed that irisin controlled glucose uptake in the skeletal muscle via the MAPK/p38 pathway, which is another probable mechanism for irisin’s protective action [52]. The MAPK/p38 pathway is thought to be an imperative inducer of NF-kB transcriptional activity, which, in turn, increases the genetic expression of proinflammatory mediators such as TNF-, MCP1, and IL-6 [52].

The antioxidant activity of irisin was reported by Mazur-Bialy etal. [53], who demonstrated that irisin significantly reduces the extensive production of harmful H_2_O_2_ by macrophages by activating the Nrf2/HO-1 pathway as well as by an increased expression of key antioxidant enzymes, including superoxide dismutase, glutathione peroxidase, and catalase 9. This finding is concomitant with our results as manifested by the marked reduction of joint tissue MDA level, along with the significant increase in PN level, in comparison with that in the CII-IFA-induced arthritic group. Likewise, irisin effectively alleviated endothelial dysfunction caused by oxidative/nitrative stress through the inhibition of PKC/NADPH oxidase and NF-kB/iNOS pathways and a reduction in the formation of peroxynitrite [54].

## 5. Conclusion

We concluded that irisin ameliorates the pathogenesis of rheumatoid arthritis in the CII-IFA experimentally induced rat model by exhibiting a remarkable inhibition of the necroptotic signaling biochemical pathway. This could be attributed to a marked suppression of TNF-α mRNA expression along with a mitigating effect of MCP1 and HMGB1 on downstream targets. In addition, it has a potent anti-inflammatory effect across decreasing proinflammatory cytokines and a marked antioxidant effect. Hence, Irisin acts as an efficient therapeutic agent for treating arthritis, as it is found to be safe with limited side effects. But still more experiments and clinical trials have to be done to prove the effects of irisin and its safe use in humans.

## 6. Limitations

To extrapolate our findings, and to have a better deducing mechanism of interplay between irisin and HMGB1, MCP1, PIPK3, and MLKL, in vitro experiments should be performed as a subsequent step to support data escalation and to pave the way for further clinical applications. Also, the use of ELISA without confirmation of the protein target is considered as one of the limitations of this study, as ELISA relies on the specificity of the antibody in the assay, and this has not been shown to specifically target the analyte target that is assumed here. This also leads to a future assessment of the interplay between the major proteins identified in this study.

## Data Availability

All data generated or analyzed during this study are included in this published article (and its supplementary information files).

## Research involving animals

All procedures performed in studies involving animals were in accordance with the ethical standards of the Animal Care Ethical Committee guidelines of the Faculty of Medicine, Tanta University.
